# Comparison of the Proliferation and Differentiation Potential of Human Urine-, Placenta Decidua Basalis-, and Bone Marrow-Derived Stem Cells

**DOI:** 10.1155/2018/7131532

**Published:** 2018-12-13

**Authors:** Chengguang Wu, Long Chen, Yi-zhou Huang, Yongcan Huang, Ornella Parolini, Qing Zhong, Xiaobin Tian, Li Deng

**Affiliations:** ^1^Laboratory of Stem Cell and Tissue Engineering, State Key Laboratory of Biotherapy, West China Hospital, Sichuan University, Chengdu 610000, China; ^2^Institute of Pathology and Molecular Pathology, University Hospital Zurich, Zurich 8091, Switzerland; ^3^Department of Orthopedics, Guizhou Provincial People's Hospital, Guiyang, Guizhou 550000, China; ^4^Shenzhen Engineering Laboratory of Orthopaedic Regenerative Technologies, Orthopaedic Research Center, Peking University Shenzhen Hospital, Shenzhen 518036, China; ^5^Shenzhen Key Laboratory of Spine Surgery, Department of Spine Surgery, Peking University Shenzhen Hospital, Shenzhen 518036, China; ^6^Department of Orthopaedics and Traumatology, The University of Hong Kong, Hong Kong SAR 999077, China; ^7^Centro di Ricerca E. Menni, Fondazione Poliambulanza Istituto Ospedaliero, Brescia 25124, Italy; ^8^Istituto di Anatomia Umana e Biologia Cellulare, Università Cattolica del Sacro Cuore Facoltà di Medicina e Chirurgia, Roma 00168, Italy; ^9^Children's Medical Research Institute, University of Sydney, Sydney, New South Wales 2145, Australia

## Abstract

Human multipotent stem cell-based therapies have shown remarkable potential in regenerative medicine and tissue engineering applications due to their abilities of self-renewal and differentiation into multiple adult cell types under appropriate conditions. Presently, human multipotent stem cells can be isolated from different sources, but variation among their basic biology can result in suboptimal selection of seed cells in preclinical and clinical research. Thus, the goal of this study was to compare the biological characteristics of multipotent stem cells isolated from human bone marrow, placental decidua basalis, and urine, respectively. First, we found that urine-derived stem cells (USCs) displayed different morphologies compared with other stem cell types. USCs and placenta decidua basalis-derived mesenchymal stem cells (PDB-MSCs) had superior proliferation ability in contrast to bone marrow-derived mesenchymal stem cells (BMSCs); these cells grew to have the highest colony-forming unit (CFU) counts. In phenotypic analysis using flow cytometry, similarity among all stem cell marker expression was found, excluding CD29 and CD105. Regarding stem cell differentiation capability, USCs were observed to have better adipogenic and endothelial abilities as well as vascularization potential compared to BMSCs and PDB-MSCs. As for osteogenic and chondrogenic induction, BMSCs were superior to all three stem cell types. Future therapeutic indications and clinical applications of BMSCs, PDB-MSCs, and USCs should be based on their characteristics, such as growth kinetics and differentiation capabilities.

## 1. Introduction

Multipotent stem cells (MSCs) are cells with broad biological function which have a unique capacity for self-renewal and display extensive multipotential for differentiation into many different cell types [1, 2], such as osteogenic, adipogenic, chondrogenic, and endothelial lineages. There are many advantages to the potential uses of MSCs. In recent years, preclinical and clinical studies have demonstrated the therapeutic potential of MSCs for vascularization [3] and regeneration of damaged tissues, such as bone, cartilage, myocardium, and tendon [4–8]. Moreover, MSCs have also shown considerable potential in the treatment of a wide spectrum of disorders such as autoimmune diseases, hematopoietic defects, and fertility preservation [9–12]. Currently, multipotent stem cells can be readily isolated from bone marrow, peripheral blood, skin, adipose tissue, urine, and placenta [4, 13–16].

Bone marrow is the most common source of multipotent stem cells. Since multipotent stem cells were first able to be isolated from bone marrow, human stem cell research has developed rapidly. For example, bone marrow-derived mesenchymal stem cells (BMSCs) have been applied to cartilage repair [5, 17, 18], intervertebral disc repair [19], and bone repair [20] in clinical practice. However, BMSCs are restricted by the invasive harvesting procedures required, which limits their use for autogenous approaches and may cause donor site morbidity [21, 22]. For these reasons, alternative sources of MSCs have been investigated.

The placenta is one alternative source of MSCs. Placenta decidua basalis-derived mesenchymal stem cells (PDB-MSCs) have drawn great interest in regenerative medicine and tissue engineering because of harvesting without invasive procedures and using without ethical concerns [23]. Some published studies have demonstrated that PDB-MSCs possess extensive capacity for self-renewal, multilineage differentiation, and considerable immunomodulatory [23, 24]. PDB-MSCs also share some properties of pluripotent embryonic stem cells as well as other properties of multipotent stem cells [16].

Recently, urine-derived stem cells (USCs) which are isolated from urine have been studied as a promising candidate for many tissue engineering therapies due to their multilineage differentiation properties (into osteocytes, chondrocytes, adipocytes, neurocyte, myocytes, and endothelial cells) and sufficient proliferation activities [13, 25, 26]. Advantages to the use of USCs include noninvasive and low-cost harvesting as well as being considered for ethical use. Additionally, USCs have been isolated from autologous urine which do not induce immune responses or rejection [25]. Therefore, USCs are considered to be an attractive alternative source of multipotent stem cells that have been appropriated for a large variety of uses.

In this study, we only focus on the differences in proliferation and differentiation potentials of USCs, PDB-MSCs, and BMSCs by comparing their morphologies, immune-phenotypes, proliferation capacities, and differentiation potentials (osteogenic, adipogenic, chondrogenic, and endothelial).

## 2. Materials and Methods

This study was approved by the Ethics Committee of West China Hospital, Sichuan University, Chengdu, China.

### 2.1. Isolation and Culture of BMSCs

Human bone marrow samples were obtained from six patients (age from 45 to 65 years old) who underwent a total hip replacement at the orthopedic department of the West China Hospital after providing written informed consent. BMSCs were isolated using the method outlined in our previous report [27]. Briefly, bone marrow aspirates were diluted with phosphate-buffered saline (PBS), layered over Ficoll solution (TBD Science, China), and centrifuged at 500 g for 30 min to collect mononuclear cells from the gradient interface. Then, mononuclear cells were cultured in the growth medium (Dulbecco's modified Eagle's medium-High Glucose (DMEM-HG, Gibco, USA) with 10% *v*/*v* fetal bovine serum (FBS, HyClone, South America) and 1% penicillin/streptomycin), which was changed to remove the nonadherent cells after 72 hours of culture. BMSCs were incubated in a T-25 culture flask at 37°C with 5% CO_2_. After reaching 70–80% confluence, cells were passaged at a dilution of 1 : 3. The 4th passage and 10th passage cells were used in the morphologic analysis, and remaining cells from the 4th passage were used in other assays.

### 2.2. Isolation and Culture of PDB-MSCs

Human placenta samples were obtained from three healthy donor mothers (age 28 to 33 years) after providing written informed consent. PDB-MSCs were then isolated from these samples according to our previous report [15, 28]. Briefly, decidua basalis was collected and washed in PBS to remove residual blood. The samples were then mechanically minced into small particles and digested with 0.25% trypsin (Gibco, USA), 0.1% collagenase IV (Invitrogen, USA), and 80 U/ml DNAse I (Sigma, USA) for 30 min at 37°C. Nucleated cells were concentrated by density gradient centrifugation (500 g for 30 min), suspended in 5 ml complete medium containing DMEM-HG (Gibco, USA) with 10% *v*/*v* FBS (HyClone, South America) and 1% penicillin/streptomycin. PDB-MSCs were incubated in a T-25 culture flask at 37°C with 5% CO_2_. After reaching 70–80% confluence, cells were passaged at a dilution of 1 : 3. The 4th passage and 10th passage cells were used in the morphologic analysis and remaining cells from the 4th passage were used in other assays.

### 2.3. Isolation and Culture of USCs

We obtained human urine samples from five healthy male adult donors (age from 24 to 30 years old) after receiving written informed consent. USCs were isolated following the protocol laid out in our previous report [25]. Briefly, urine samples with penicillin and streptomycin were centrifuged at 500 g for 10 min at room temperature. The cell pellets were washed with PBS (pH = 7.4) and centrifuged again for 10 minutes at 500 g. To determine the total number of living cells shed into the urine, cells were stained with trypan blue and counted. Then, cells were finally seeded in 24-well plates with culture medium (keratinocyte serum-free medium and progenitor cell medium at a ratio of 1 : 1 as well as 5% *v*/*v* FBS (HyClone, South America) as described by Chen et al. [25] and Zhang et al. [29]). USCs were incubated in a T-25 culture flask at 37°C with 5% CO_2_. After reaching 70–80% confluence, cells were passaged at a dilution of 1 : 3. The 4th passage and 10th passage cells were used in the morphologic analysis, and remaining cells from the 4th passage were used in other assays.

### 2.4. Colony-Forming Unit (CFU) Assay

Colony-forming ability analysis was modified from our previous study [15]. BMSCs, PDB-MSCs, and USCs were plated at a density of 80 cells/cm^2^ and cultured in complete medium (H-DMEM with 10% FBS for BMSCs and PDB-MSCs; USC culture medium for USCs). After 8 days, cells were fixed with methanol and stained with 0.1% crystal violet (Sigma, USA) solution for 30 min at 37°C. We calculated the number of MSC clones by using the Image-Pro Plus 6.0 software (Media Cybernetics, USA), and a collection of more than 50 cells was counted as one colony [30].

### 2.5. Cell Proliferation

Cell proliferation was assessed according to our previous study method [15]. Briefly, 2 × 10^3^ BMSCs, PDB-MSCs, and USCs were plated in a 96-well plate (*n* = 6). Cell viability was monitored on days 0, 1, 3, 5, 7, and 9, respectively. One hour after the addition of 10 *μ*l CCK-8 (Dojindo, Japan) in 100 *μ*l culture medium, optical density was determined using a spectrophotometer at 490 nm with background correction at 630 nm.

### 2.6. Flow Cytometry Analysis

The 4th passage of BMSCs, PB-MSCs, and USCs was harvested by 0.25% trypsin/ethylenediaminetetraacetic acid (EDTA). For each staining, about 1 × 10^6^ cells were incubated with fluorescein isothiocyanate (FITC) or phycoerythrin- (PE-) conjugated monoclonal antibodies: CD29, CD34, CD45, CD73, CD90, CD105, CD166, and HLA-DR (BD Pharmingen™) at 4°C for 30 min in the dark room and then washed and resuspended in 200 *μ*l PBS (pH = 7.4). The phenotypic analysis was performed with a Beckman Cytomics FC 500 Flow Cytometry Analyzer (Beckman Coulter, USA). CD31 expression after endothelial differentiation was tested under the same procedure.

### 2.7. *In Vitro* Differentiation

In order to analyze the multipotency of PB-MSCs, osteogenic, adipogenic, chondrogenic, and endothelial differentiation was performed in specific induction media. Osteogenic and adipogenic differentiation methods were optimized from our previous study [15].

#### 2.7.1. Osteogenic Induction

In order to induce osteogenic differentiation, BMSCs, PDB-MSCs, and USCs were seeded in 6-well plates at a density of 3 × 10^4^ cells/well and cultured with osteogenic medium [15] for 21 days with the medium changed every 3 days. After induction, cells were fixed and stained with Alizarin Red solution (Sigma, USA) for 30 min. To quantify the positively stained areas after osteogenic induction, ImageJ software (National Institutes of Health, USA, version 1.47t) was used. Red staining was detected by filtering by color thresholds (L^∗^ 0-200 for Alizarin Red staining) in the Lab color space. Fixed thresholds were used for each set of images. The ratio of positive pixels to the total number of pixels per image was quantified.

#### 2.7.2. Adipogenic Induction

For adipogenic induction, BMSCs, PDB-MSCs, and USCs were plated at a density of 3 × 10^4^ cells/well and induced in the adipogenic medium [15]. The medium was replaced every 3 days. After 8 days, cells were fixed and stained with Oil Red O solution (Sigma, USA) for 30 min to visualize lipid vacuoles. In order to quantify the positively stained areas after adipogenic induction, ImageJ software (National Institutes of Health, USA, version 1.47t) was used. Red staining was detected using color thresholds (L^∗^ 0-230 for Oil Red O staining) in the Lab color space. Fixed thresholds were used for each set of images. The ratio of positive pixels to the total number of pixels per image was calculated.

#### 2.7.3. Chondrogenic Induction

Chondrogenic differentiation was performed following the protocol described in our previous study [25]. Briefly, BMSCs, PDB-MSCs, and USCs were centrifuged for 5 min at 500 g with a density of 1 × 10^6^ cells after which the pellet was cultured in chondrogenic medium (Cyagen Biosciences Inc., USA). The medium was replaced every 2–3 days. After 21 days, the pellets were fixed with 4% paraformaldehyde for 24 h and washed in PBS three times. Then, the fixed pellets were taken out, washed with 50% and 70% ethanol for dehydration, embedded in paraffin, and sectioned. Deparaffinized sections were stained with Toluidine Blue solution for 30 min to visualize extracellular matrix-bound proteoglycans.

#### 2.7.4. Endothelial Induction and Tube Formation Assay

For endothelial differentiation, BMSCs, PDB-MSCs, and USCs have plated at a density of 3 × 10^4^ cells/well and induced in endothelial basal medium (EBM2, Lonza) supplemented with 50 ng/ml vascular endothelial growth factor (VEGF, PeproTech, USA). The medium was then replaced every 3 days. After 8 days, cells were collected for CD31 expression analysis, tube formation assay, and real-time PCR testing.

*In vitro* tube formation assay was conducted in order to further investigate endothelial differentiation function. Nontreated and endothelial-induced BMSCs, PDB-MSCs, and USCs were seeded on Matrigel in 48-well dishes at a density of 6 × 10^4^ cells/well. Human umbilical vein endothelial cells (HUVECs) served as a positive control. After 20 hours, cells were rinsed with PBS followed by staining Hoechst 33342 (Sigma, USA) solution (10 *μ*g/ml) for 30 min in the incubator at 37°C with 5% CO_2_. A fluorescence microscope (Olympus IX50, Japan) was used for the analysis of network formation. The number of tubes was counted using the Image-Pro Plus 6.0 software (Media Cybernetics, USA).

### 2.8. Quantitative Reverse-Transcriptase–Polymerase Chain Reaction (RT-PCR)

RT-PCR was performed to analyze the endothelium-related gene expression after endothelial induction for 8 days and chondrogenic cell-related gene expression after chondrogenic induction for 21 days. Total RNA was extracted to use RNAiso Plus reagent (Takara, Japan) and reverse-transcribed into cDNA to use a PrimeScript RT reagent Kit (Takara, Japan). The expression of specific genes was quantified to use SYBR Premix Ex Taq II kit (Takara, Japan) in an IQ5 real-time system (Bio-Rad, USA). The specific primers for endothelial-related genes (von Willebrand factor (vWF) and PECAM-1) and chondrogenic cells had related genes (hCOL2A1 and hACANF) which are presented in Table 1. Each target gene expression was analyzed and compared to the housekeeping gene glyceraldehyde-3-phosphate dehydrogenase (GAPDH). Data were analyzed by the 2^-△△Ct^ method, and results were expressed relative to the gene expression level of the BMSC control group.

### 2.9. Statistical Analysis

All data are expressed as mean ± SD. Statistical analysis was performed to use SPSS 17.0 software (SPSS, USA). Results were analyzed with Student's *T*-test, and *P* < 0.05 was considered statistically significant.

## 3. Results

### 3.1. Morphology

**Figure 1:**
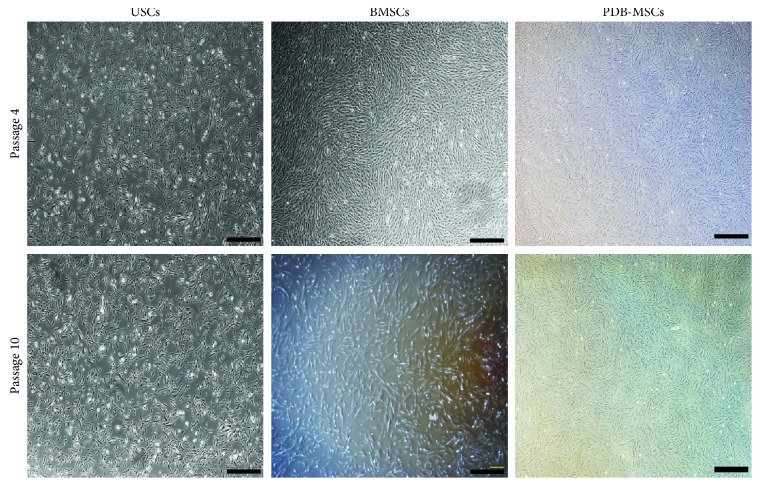
USC, BMSC, and PDB-MSC morphology at passage 4 and 10. Scale bar = 500 *μ*m. BMSCs: bone marrow-derived mesenchymal stem cells; PDB-MSCs: placenta decidua basalis derived mesenchymal stem cells; USCs: urine-derived stem cells.

After culturing for 7 to 10 days, adherent cells from bone marrow, urine, and placenta digestion began to form cell clones. These clonogenic cells reached 70 to 80% confluence within 2 weeks. BMSCs and PDB-MSCs displayed similar spindle-shaped fibroblast-like morphology, and USCs had a cobble stone-like shape with frills after passage 4 and passage 10 (Figure 1).

### 3.2. CFUs, Proliferation, and Phenotype

**Figure 2:**
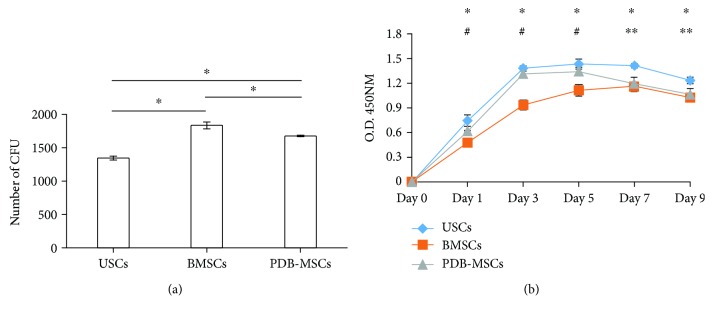
Results of colony-forming unit (CFU) assay and cell proliferation analysis. (a) Quantification of stem cells colony-forming units (CFU) (^∗^*P* < 0.05). (b) Proliferation curve of three tested stem cells (^∗^*P* < 0.05, USC versus BMSCs; ^#^*P* < 0.05, PDB-MSCs versus BMSCs; and ^∗∗^*P* < 0.05, USC versus PDB-MSCs). BMSCs: bone marrow-derived mesenchymal stem cells; PDB-MSCs: placenta decidua basalis-derived mesenchymal stem cells; USCs: urine-derived stem cells; CFU: colony-forming unit.

In colony formation unit analysis, BMSCs demonstrated much better colony-forming abilities compared with USCs and PDB-MSCs; PDB-MSCs resulted in better colony-forming abilities than USCs (BMSCs = 1820 ± 67, PDB − MSCs = 1660 ± 32, and USCs = 1330 ± 45, *P* < 0.05) (Figure 2(a)). As measured by trypan blue exclusion, the average total number of living cells in the pellet derived from the 200 ml urine sample was 784 ± 180.8. The number of the clone forming units of primary USCs derived from the 200 ml urine sample was ranging from 10 to 18.

In comparison to cell proliferation, PDB-MSCs and USCs both reached peak growth speed on day 3 and presented higher proliferative capacities in contrast to BMSCs from day 1 to day 5. During this period, no significant difference was been found between USCs and PDB-MSCs (Figure 2(b)). USCs had the highest growth rate from day 7 to day 9. During this period, no significant difference was been found between PDB-MSCs and BMSCs (Figure 2(b)).

**Figure 3:**
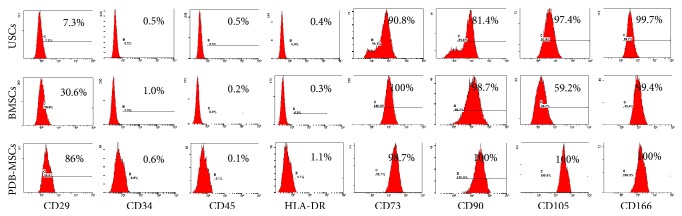
Immunophenotype of BMSCs, PDB-MSCs, and USCs by flow cytometry. BMSCs: bone marrow-derived mesenchymal stem cells; PDB-MSCs: placenta decidua basalis-derived mesenchymal stem cells; USCs: urine-derived stem cells.

Phenotypic analysis was performed using flow cytometry (Figure 3). All three cell types revealed similar negative expression for CD34, CD45, and HLA-DR (below 1%), but they exhibited different trends in terms of CD29 (USCs 7.3%, BMSCs 30.6%, and PDB-MSCs 86%). Notably, even though the expressions of CD73, CD90, CD105, and CD166 are all positive among the three tested cells (most of them were above 95%), BMSCs were weakly positive for CD105 (59.2%).

### 3.3. Multilineage Differentiation Potential

**Figure 4:**
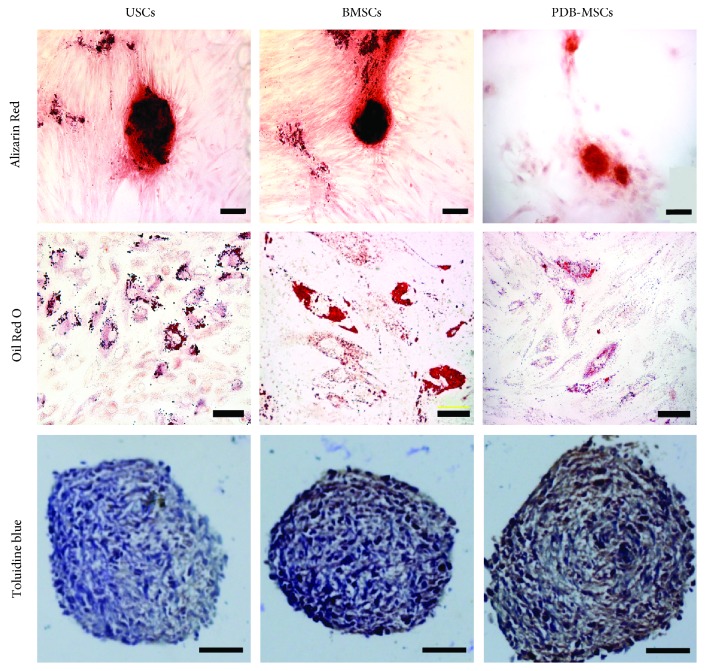
Osteogenic, adipogenic, and chondrogenic differentiation of USCs, BMSCs, and PDB-MSCs stained, respectively, with Alizarin Red (scale bar = 100 *μ*m), Oil Red O (scale bar = 20 *μ*m), and Toluidine Blue (scale bar = 50 *μ*m). BMSCs: bone marrow-derived mesenchymal stem cells; PDB-MSCs: placenta decidua basalis-derived mesenchymal stem cells; USCs: urine-derived stem cells.

The multilineage differentiation ability of USCs, BMSCs, and PDB-MSCs was analyzed *in vitro*. Under conditioned culture medium for certain days, all the cells were positively stained for Alizarin Red, Oil Red O, and Toluidine Blue, which demonstrated their multilineage differentiation potential (Figure 4). Their differentiation capabilities, however, were different with respect to the positive staining area as evaluated by ImageJ and RT-PCR results.

**Figure 5:**
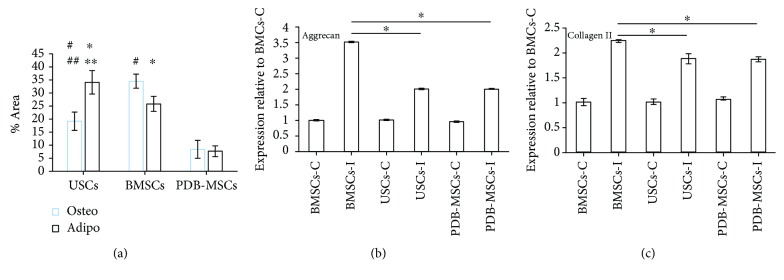
Quantitative studies of the osteogenic, adipogenic, and chondrogenic differentiation abilities of USCs, BMSCs, and PDB-MSCs. (a) Alizarin Red- and Oil Red O-positive staining area analyzed by ImageJ (^#^*P* < 0.05, compared to PDB-MSCs for osteogenic differentiation comparison; ^##^*P* < 0.05, compared to BMSCs for osteogenic differentiation comparison; ^∗^*P* < 0.05, compared to PDB-MSCs for adipogenic differentiation comparison; and ^∗∗^*P* < 0.05, compared to BMSCs for adipogenic differentiation comparison). (b) mRNA expression of aggrecan quantitated in USCs, BMSCs, and PDB-MSCs after 21 days of induction (^∗^*P* < 0.05). (c) mRNA expression of collagen II was quantitated in USCs, BMSCs, and PDB-MSCs after 21 days of chondrogenic induction (^∗^*P* < 0.05). BMSCs: bone marrow-derived mesenchymal stem cells; PDB-MSCs: placenta decidua basalis-derived mesenchymal stem cells; USCs: urine-derived stem cells; C: control; I: introduced.

For osteogenic differentiation (Figure 5(a)), the positive area of BMSCs was 34.5% ± 2.7%, which was superior to USCs and PDB-MSCs (19.2% ± 3.5% and 8.4% ± 3.4%, *P* < 0.05). USCs also resulted in a larger positive area compared to PDB-MSCs (*P* < 0.05).

For adipogenic differentiation (Figure 5(a)), the positive areas of USCs, BMSCs, and PDB-MSCs were 34.1% ± 4.5%, 25.8% ± 2.9%, and 7.7% ± 2.1%, respectively. The positive areas in USC images were larger than for BMSC and PDB-MSC images (*P* < 0.05) while BMSCs had larger positive areas than PDB-MSCs (*P* < 0.05).

For chondrogenic differentiation (Figures 5(b) and 5(c)), the results of RT-PCR indicated that the expression of chondrogenesis-related genes (aggrecan and collagen II) in induced BMSCs were significantly higher than induced USCs as well as PDB-MSCs. Induced USCs were similar to induced PDB-MSCs at the level of aggrecan and collagen II gene expression.

**Figure 6:**
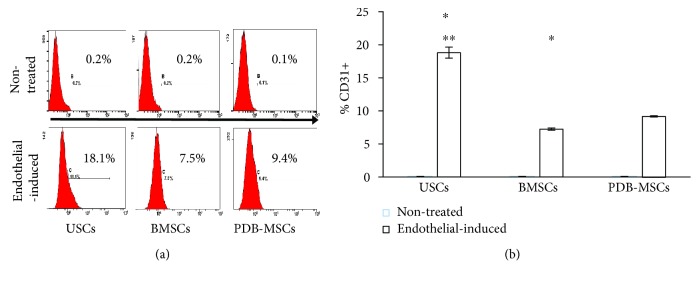
CD31 expression in USCs, BMSCs, and PDB-MSCs, after 8 days of endothelial induction. (a) Flow cytometry analysis of CD31 expression in USCs, BMSCs, and PDB-MSCs after 8 days of endothelial induction. (b) Quantitative evaluation of CD31 expression (^∗^*P* < 0.05, compared to PDB-MSCs; ^∗∗^*P* < 0.05, compared to BMSCs). BMSCs: bone marrow-derived mesenchymal stem cells; PDB-MSCs: placenta decidua basalis-derived mesenchymal stem cells; USCs: urine-derived stem cells.

In differentiation analysis of endothelial cells, nontreated groups exhibited negative results with CD31 expression under 0.2% and endothelial-induced USCs, BMSCs, and PDB-MSCs had 18.8% ± 0.80%, 7.23% ± 0.30%, and 9.16% ± 0.25% expression, respectively (Figure 6(a)). CD31 expression of endothelial-induced USCs is the highest among three endothelial-induced groups (*P* < 0.05). Endothelial-induced PDB-MSCs resulted in higher CD31 expression than endothelial-induced BMSCs (Figure 6(b)).

### 3.4. Tube Formation Assay

**Figure 7:**
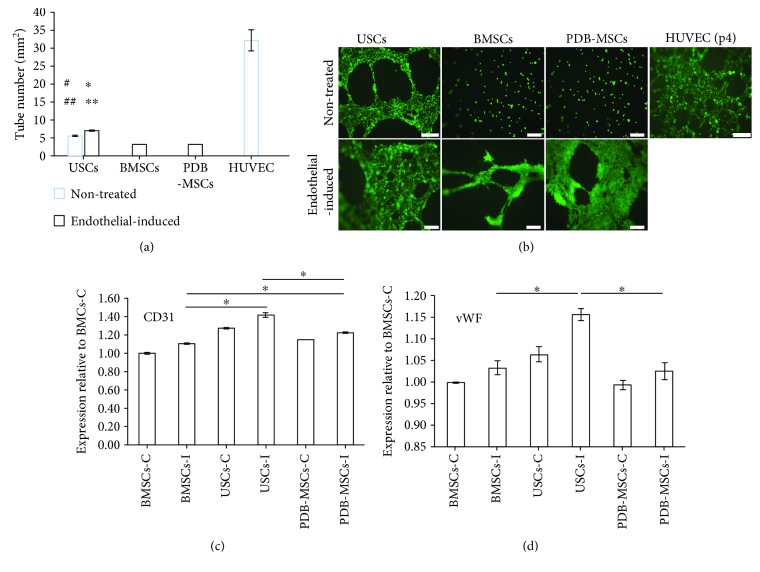
Results of tube formation assay. (a) Quantitative evaluation of formed tube number in the USC, BMSC, and PDB-MSC groups, respectively (^#^*P* < 0.05, compared to BMSCs for nontreated group comparison; ^##^*P* < 0.05, compared to PDB-MSCs for nontreated group comparison; ^∗^*P* < 0.05, compared to BMSCs for endothelial differentiated group comparison; and ^∗∗^*P* < 0.05, compared to PDB-MSCs for endothelial differentiated group comparison). (b) Image of endothelial differentiated USCs, BMSCs, and PDB-MSCs and untreated cells cultured on Matrigel for 20 hours to form branched networks (angiogenesis) and tubular structures (scale bar = 200 *μ*m); (c, d) mRNA expression of CD31 and vWF in USCs, BMSCs, and PDB-MSCs quantitated after 21 days of endothelial induction (^∗^*P* < 0.05). BMSCs: bone marrow-derived mesenchymal stem cells; PDB-MSCs: placenta decidua basalis-derived mesenchymal stem cells; USCs: urine-derived stem cells; C: control; I: introduced; HUVEC: human umbilical vein endothelial cells; vWF: von Willebrand factor.

In addition, we performed tube formation assay to assess stem cell vascularization potential. All the endothelial differentiated groups exhibited *in vitro* “vessel” formation on Matrigel after 20 hours (number of tubes/mm^2^: USC = 7.07 ± 0.25, BMSC = 3.00 ± 0.03, and PDB − MSC = 3.22 ± 0.03). Endothelial-induced USCs had a higher number of tubes/mm^2^ than endothelial-induced BMSCs and PDB-MSCs (*P* < 0.05). There was no significant difference between endothelial-induced PDB-MSCs and BMSCs in number of tubes/mm^2^. Within the nontreated groups, no tubes were observed of BMSCs and PDB-MSCs. Intriguingly, even without induction, the USC group (the number of tubes/mm^2^: 5.61 ± 0.49) appeared to have similar tube forming ability as treated groups. Quantitative results demonstrated that USCs had the highest tube number per millimeter square (*P* < 0.05) in both treated and nontreated groups (Figures 7(a) and 7(b)).

Lastly, gene expression levels of two endothelial cell markers, CD31 and vWF, were all enhanced in three groups after induction and the expression levels of differentiated USCs were significantly higher than in the other two groups (*P* < 0.05). Endothelial induced PDB-MSCs resulted in higher CD31 gene expression levels than endothelial induced BMSCs (*P* < 0.05). There was no significant difference between endothelial induced PDB-MSCs and BMSCs in vWF gene expression level (Figures 7(c) and 7(d)).

## 4. Discussion

Human stem cells harvested from different tissues have distinct features, including cell proliferation, colony formation ability, and differentiation capability. Thus, it is essential to obtain identifying biophysical information about each cell type in order to optimize experimental and clinical selection of seed cells. Here for the first time, the bio-characteristics of stem cells derived from human bone marrow, placenta decidua basalis, and urine were investigated, specifically cell morphology, proliferation, phenotype, and multilineage differentiation properties. Although this study did not include other common stem cell candidates, comparison of the most promising or most widely used seed cells including USCs, BMSCs, and PDB-MSCs might provide valuable evidence for stem cell transplantation research.

Morphologically, BMSCs and PDB-MSCs have displayed spindle-shaped and fibroblast-like morphology, which is considered to be a typical character of mesoderm-origin mesenchymal stem cells while USCs had a rice-grain-like shape. This urothelial-like shape was consistent with recent evidence from other groups [13, 25]. Moreover, the morphology of BMSCs and PDB-MSCs is more elongated and dispersed. The efficiency to form colonies still remains an important assay for the quality of cell preparations [31]. The CFU assay in this study indicates that BMSCs, PDB-MSCs, and USCs showed the abilities of colony forming. However, BMSCs have resulted in better colony-forming abilities than PDB-MSCs and USCs.

In our cell proliferation assay, USCs and PDB-MSCs showed better proliferation ability than BMSCs during the early stage of proliferation test (days 1–5). At the late stage of the proliferation test, USCs showed better proliferation ability than did PDB-MSCs and BMSCs (days 7–9). The growth curves revealed a better proliferation ability of USCs than those of PDB-MSCs or BMSCs did during the entire proliferation test (days 1–9). Despite no previous direct comparison study of these three cell types, previous research predicted our findings as BMSCs seemed to be inferior to other multipotent stem cells regarding the growth kinetics [14]. Proliferation and CFU assays were performed using different standard culture media specific to BMSCs, PDB-MSCs, and USCs. Therefore, CFU and proliferation assays allowed comparing stem cell proliferation and stemness capabilities using optimized cell culture conditions.

As part of our phenotypic investigation, flow cytometry used to detect all three cell types tested did not express hematopoiesis-related antigens CD34 and CD45 (below 2%); human leukocyte antigen HLA-DR is also negative (below 2%). Meanwhile, each cell type had positively expressed mesenchymal stem cell-related antigens (CD73, CD90, CD105, and CD166). These findings were in agreement with the minimal experimental criteria for mesenchymal stem cells as proposed by the International Society for Cellular Therapy [32]. Furthermore, marked differences of CD29 and relatively low CD105 expression of BMSCs were observed; these findings appeared to be inconsistent with some literatures. A great variety might be caused by stem cell origin, harvesting procedure, and subpopulations [13, 14].

Elucidation of the multilineage differentiation capability of USCs, BMSCs, and PDB-MSCs will lead to a better understanding of their biological roles. Our data indicated that all the multipotent stem cells, in our investigation, possessed multilineage differentiation properties. According to our quantitative results, BMSCs had better osteogenic and chondrogenic abilities than USCs and PDB-MSCs while USCs had better adipogenic and endothelial abilities than BMSCs or PDB-MSCs. Recent evidence from the literature demonstrates that multipotent stem cells have held a preferential differentiation trend into their tissue of origin, as the BMSCs had the best osteogenic and chondrogenic differentiation potential and the USCs and PDB-MSCs showed relatively low expression of osteogenic and chondrogenic makers, which may be regulated by their epigenetic status [33]. Surprisingly, we also found that USCs harbored robust endothelial differentiation abilities even without induction conditions. Further research is needed to understand these underlying mechanisms.

The common strategy to overcome the lack of vascularization in tissue engineering is based on the endothelial cells and their ability to form new vessels, a process known as neoangiogenesis [34]. In this study, tube formation assay was performed to assess the vascularization potential of BMSCs, USCs, and PDB-MSCs. As the results above describe, USCs had the best endothelial cell differentiation capability among the three investigated sources of stem cells. Endothelial-induced USCs had a higher number of tubes/mm^2^ than endothelial induced BMSCs and PDB-MSCs. Previous studies [13, 35, 36] have demonstrated that there is strong evidence that USCs are most likely from glomerular parietal epithelial cells in kidney, which also could explain why USCs have the best endothelial differentiation capacity. Taken together, these findings revealed that USC finding could be an alternative cell source for tissue engineering with better endothelial cell differentiation capability and vascularization potential than BMSCs and PDB-MSCs.

## 5. Conclusion

In summary, this study demonstrates that USCs have different morphologies compared with the other stem cell types. USCs and PDB-MSCs both showed better proliferation ability than BMSCs. However, BMSCs had better colony-forming abilities than PDB-MSCs and USCs. In a phenotypic analysis using flow cytometry, similarity of stem cell marker expression was found excluding CD29 and CD105. In addition, BMSCs were observed to have better osteogenic and chondrogenic abilities than USCs and PDB-MSCs. USCs had better adipogenic and endothelial abilities as well as vascularization potential than BMSCs and PDB-MSCs. Future therapeutic indications and clinical applications of BMSCs, PDB-MSCs, and USCs should be designed based on three unique characteristics among different subtypes, such as their growth kinetics and differentiation capabilities.

## Figures and Tables

**Table 1:** Primer sequences (5′-3′) used in real time RT-PCR.

Gene	Primer sequences
GAPDH	GTGGACCTGACCTGCCGTCT (F)
	GGAGGAGTGGGTGTCGCTGT (R)
vWF	TAGAATCCTTACCAGTGACG (F)
	ACTCACACTCATACCCGTTC (R)
PECAM-1	GCTGACCCTTCTGCTCTGTT (F)
	TGAGAGGTGGTGCTGACATC (R)
hCOL2A1	GCTCCCAGAACATCACCTACC (F)
	CAGTCTTGCCCCACTTACCG (R)
hACAN	GCCTATCAGGACAAGGTCTCAC (F)
	ATGGCTCTGTAATGGAACACGA (R)

## Data Availability

The data used to support the findings of this study are available from the corresponding author upon request.
