# Corrigendum to “Comparison of the Proliferation and Differentiation Potential of Human Urine-, Placenta Decidua Basalis-, and Bone Marrow-Derived Stem Cells”

**DOI:** 10.1155/2019/1651506

**Published:** 2019-03-10

**Authors:** Chengguang Wu, Long Chen, Yi-zhou Huang, Yongcan Huang, Ornella Parolini, Qing Zhong, Xiaobin Tian, Li Deng

**Affiliations:** ^1^Laboratory of Stem Cell and Tissue Engineering, State Key Laboratory of Biotherapy, West China Hospital, Sichuan University, Chengdu 610000, China; ^2^Institute of Pathology and Molecular Pathology, University Hospital Zurich, Zurich 8091, Switzerland; ^3^Department of Orthopedics, Guizhou Provincial People's Hospital, Guiyang, Guizhou 550000, China; ^4^Shenzhen Engineering Laboratory of Orthopaedic Regenerative Technologies, Orthopaedic Research Center, Peking University Shenzhen Hospital, Shenzhen 518036, China; ^5^Shenzhen Key Laboratory of Spine Surgery, Department of Spine Surgery, Peking University Shenzhen Hospital, Shenzhen 518036, China; ^6^Department of Orthopaedics and Traumatology, The University of Hong Kong, Hong Kong SAR 999077, China; ^7^Centro di Ricerca E. Menni, Fondazione Poliambulanza Istituto Ospedaliero, Brescia 25124, Italy; ^8^Istituto di Anatomia Umana e Biologia Cellulare, Università Cattolica del Sacro Cuore Facoltà di Medicina e Chirurgia, Roma 00168, Italy; ^9^Children's Medical Research Institute, University of Sydney, Sydney, New South Wales 2145, Australia

In the article titled “Comparison of the Proliferation and Differentiation Potential of Human Urine-, Placenta Decidua Basalis-, and Bone Marrow-Derived Stem Cells” [1], there were the following errors:

- The first image (USCs) in the second row (passage 10) in Figure 1 was incorrect
- There was a missing in-text citation for reference [15] in the legend of Figure 4

The corrected Figure 1 and the corrected legend of Figure 4 along with the figure itself are shown herein.

## Figures and Tables

**Figure 1:**
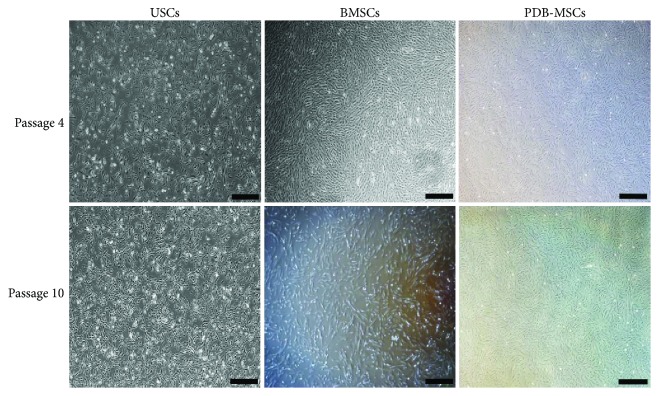
USC, BMSC, and PDB-MSC morphologies at passages 4 and 10. Scale bar = 500 *μ*m. BMSCs: bone marrow-derived mesenchymal stem cells; PDB-MSCs: placenta decidua basalis-derived mesenchymal stem cells; USCs: urine-derived stem cells.

**Figure 2:**
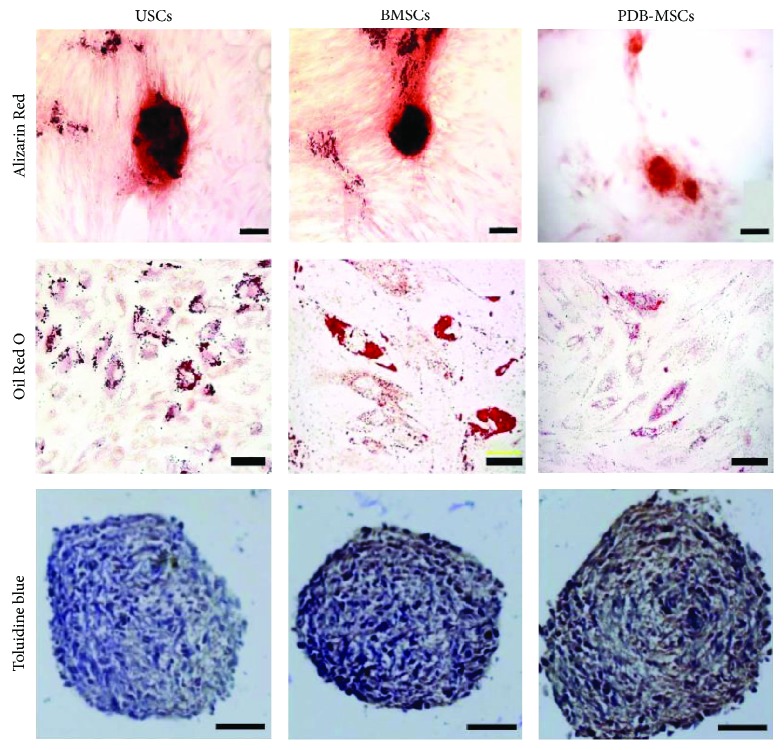
Osteogenic, adipogenic [15], and chondrogenic differentiation of USCs, BMSCs, and PDB-MSCs stained with Alizarin Red (scale bar = 100 *μ*m), Oil Red O (scale bar = 20 *μ*m), and Toluidine Blue (scale bar = 50 *μ*m), respectively. BMSCs: bone marrow-derived mesenchymal stem cells; PDB-MSCs: placenta decidua basalis-derived mesenchymal stem cells; USCs: urine-derived stem cells.
